# (−)-β-Caryophyllene, a CB2 Receptor-Selective Phytocannabinoid, Suppresses Motor Paralysis and Neuroinflammation in a Murine Model of Multiple Sclerosis

**DOI:** 10.3390/ijms18040691

**Published:** 2017-04-01

**Authors:** Thaís Barbosa Alberti, Wagner Luiz Ramos Barbosa, José Luiz Fernandes Vieira, Nádia Rezende Barbosa Raposo, Rafael Cypriano Dutra

**Affiliations:** 1 Laboratory of Autoimmunity and Immunopharmacology (LAIF), Department of Health Sciences, Center of Araranguá, Federal University of Santa Catarina, Araranguá 88906-072, Brazil; alberti.thais@gmail.com; 2 Faculty of Pharmaceutical Sciences, Federal University of Pará, Belém 66075-740, Brazil; zweigw@gmail.com (W.L.R.B.); jvieira@ufpa.br (J.L.F.V.); 3 Research and Innovation in Health Sciences (NUPICS), Federal University of Juiz de Fora, Juiz de Fora 36036-900, Brazil; nadiacritt@gmail.com

**Keywords:** multiple sclerosis, neuroinflammation, experimental autoimmune encephalomyelitis, CB2 receptor, (−)-β-caryophyllene, phytocannabinoid

## Abstract

(−)-β-caryophyllene (BCP), a cannabinoid receptor type 2 (CB2)-selective phytocannabinoid, has already been shown in precedent literature to exhibit both anti-inflammatory and analgesic effects in mouse models of inflammatory and neuropathic pain. Herein, we endeavored to investigate the therapeutic potential of BCP on experimental autoimmune encephalomyelitis (EAE), a murine model of multiple sclerosis (MS). Furthermore, we sought to demonstrate some of the mechanisms that underlie the modulation BCP exerts on autoimmune activated T cells, the pro-inflammatory scenery of the central nervous system (CNS), and demyelination. Our findings demonstrate that BCP significantly ameliorates both the clinical and pathological parameters of EAE. In addition, data hereby presented indicates that mechanisms underlying BCP immunomodulatory effect seems to be linked to its ability to inhibit microglial cells, CD4+ and CD8+ T lymphocytes, as well as protein expression of pro-inflammatory cytokines. Furthermore, it diminished axonal demyelination and modulated Th1/Treg immune balance through the activation of CB2 receptor. Altogether, our study represents significant implications for clinical research and strongly supports the effectiveness of BCP as a novel molecule to target in the development of effective therapeutic agents for MS.

## 1. Introduction

Multiple sclerosis (MS) is a severe inflammatory demyelinating disease of the central nervous system (CNS), and is the most common cause of non-traumatic neurologic disability in young adults, affecting over two million people worldwide. The etiology of MS is heterogeneous in nature and has not been completely elucidated. However, studies with MS patients suggest that the demyelination observed in the CNS results from a T cell-mediated autoimmune response, especially encephalitogenic Th17 and Th1 cells [1]. Moreover, experimental autoimmune encephalomyelitis (EAE) is an experimental model extensively employed for studying clinical, immunological, and neuropathological features of MS [2]. The onset and development of clinical signs in EAE are related to the disruption of blood–brain barrier (BBB) integrity though the upregulated expression of adhesion molecules in CNS tissues, resulting in the passage of peripheral inflammatory mediators into the CNS [3,4]. In mice, EAE is induced by triggering an immunological response when injected with myelin antigens, such as proteolipid protein peptide (PLP) and myelin oligodendrocytes glycoprotein (MOG) [5]. Th1 and Th17 cytokines have been found in CNS inflammatory lesions, whilst Th2 cytokines are absent, which suggests that Th1/Th17 cytokines play a critical part in the disease’s pathogenesis [6,7,8]. Presently, there is no cure for demyelinating diseases and their progression, and since symptoms tend to vary within the patients, seeking early treatment is essential. In general, treatment focuses on: (i) managing clinical manifestations; (ii) attenuating effects of the attacks; and (iii) altering the course of the disease. Currently, new drugs for demyelinating diseases human and/or animal, including MS, have been made available. Nonetheless, most of the current therapies possess limitations related to severe side effects and, most importantly, do not efficaciously alter the course of the disease [9,10,11]. Thus, further therapeutic alternatives are imperatively in demand. In order to limit disease progression, the therapeutic agent is required to provide efficient and early immunomodulation to counterpoise the biochemical cascade which eventually culminates in neurodegeneration and irreversible disability.

(−)-β-caryophyllene (BCP), a functional non-psychoactive CB2 receptor agonist [12,13,14], is found in, for example, plant-derived oleoresins, essential oils, solutes, distillates, extracts, fermentations, infusions, and leachates, from several plants [15,16,17,18,19,20,21,22,23,24,25]. Furthermore, BCP showed analgesic, anti-inflammatory, neuroprotective, anti-depressive, anxiolytic, and anti-nephrotoxicity effects [12,13,26,27,28,29,30,31]. In addition, several studies support the evidence that cannabimimetic drugs, mainly CB2 ligands, might have therapeutic potential in numerous pathologies, including demyelinating diseases [32,33,34,35]. Cannabinoids, including phytocannabinoids, have the ability to downregulate leucocytes proliferation, promote both T cells and macrophages apoptosis, and also diminish secretion of pro-inflammatory cytokines. This confers to cannabinoids potent inflammatory and immunomodulatory activity. In fact, in different animal studies, in vivo and in vitro, they have exhibited immunosuppressive effects [34]. Likewise, in the last decade, an increasing number of pre- and clinical trials of cannabinoids have been undertaken, mainly regarding the regulation of spasticity in multiple sclerosis [36].

As with most neurological drugs, incremental dose treatment is fundamental to reach optimum effect and to minimize side effects. Medicinal grade cannabinoids are available, however, license and usage regulations vary from country to country, and include: nabilone, dronabinol, and nabiximol [36]. Thus, the role of CB2 receptor agonists in general, and BCP in particular, in the prevention and treatment of demyelinating diseases, has not previously been studied. In this research work, we investigated the role of BCP on EAE disease progression, as well as determined the mechanisms underlying the modulation of the inflammatory circumstances of the CNS during experimental MS, in order to ascertain its therapeutic potential.

## 2. Results and Discussion

### 2.1. BCP Anti-Inflammatory Effects Are Mediated by Modulation of CB2 Receptor in MOG-Primed T Cells

**Figure 1:**
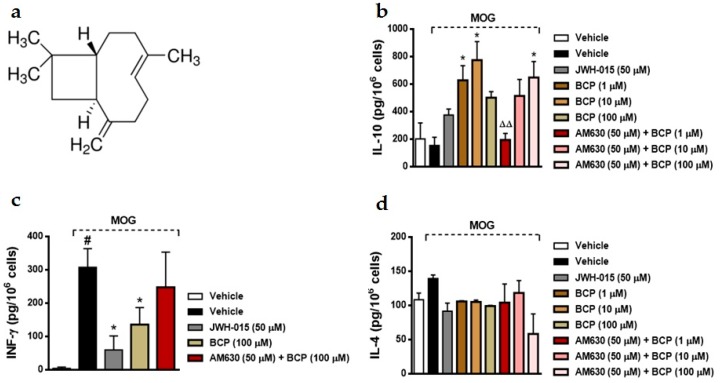
(−)-β-caryophyllene (BCP) treatment modulates Ag-specific Th1 vs. Treg cell immune responses. (**a**) Chemical structure of the sesquiterpene BCP (1*R*,4*E*,9*S*)-4,11,11-trimethyl-8-methylidenebicyclo[7.2.0]undec-4-ene. The cytokine level was assayed by ELISA—1 × 10^6^ cells. Lymphocytes were extracted from experimental autoimmune encephalomyelitis (EAE) mice on day 15 post-immunization and stimulated in the absence or presence of myelin oligodendrocytes glycoprotein (MOG) 35–55 peptide (10 μg/mL). IL-10 (**b**), IFN-γ (**c**), and IL-4 (**d**) protein level was evaluated in cells treated with the cannabinoid 2 receptor (CB2) selective agonist JWH-015 (2-methyl-1-propyl-1H-indol-3-yl)-1-naphthalenylmethanone; 50 μM), (−)-β-caryophyllene (1–100 μM), or the combination of CB2 selective antagonist AM630 (6-iodo-2-methyl-1-[2-(4-morpholinyl)ethyl]-1H-indol-3-yl-(4-methoxyphenyl)-methanone). Drugs were added 1 h before Ag-specific, and AM630 was administered 30 min before (−)-β-caryophyllene treatment. Data represents the mean ± SEM (*n* = 5 animals). ^#^ *p* < 0.05 vs. vehicle untreated cells, * *p* < 0.05 vs. EAE-untreated cells and stimulated with MOG35-55 group, ^ΔΔ^ *p* < 0.001 vs. the (−)-β-caryophyllene–treated group (one-way ANOVA followed by Newman–Keuls post hoc test).

Multiple sclerosis (MS) is a challenging CNS disorder that seems to be induced by a shift in immune response, leading to excess inflammation causing demyelination and axonal loss. Moreover, different therapeutic strategies have targeted antigen-presenting cells (APCs), B cells, T cells (Th1/Th2/Th17/T regulatory (Treg) cells), and cytokine networks associated with the demyelinating process [37]. Agonism of CB1 and CB2 receptors by exogenous ligands or endocannabinoids in animal models of MS have demonstrated suppression of pro-inflammatory mediators [38], as well as induction of neuroprotection and immunomodulation [39,40,41,42]. BCP (Figure 1), a dietary functional non-psychoactive CB2 receptor ligand [12,13,14] showed analgesic, anti-inflammatory, anticonvulsant, and neuroprotective effects [15,27,29,43,44,45,46]. Nonetheless, the effects of BCP on immune inflammatory diseases of the CNS, such as MS, have not yet been reported. Here, initially we examine the effect of BCP on both antigen (Ag) specific and nonspecific lymphocytes involved in EAE pathogenesis. On day 15 post-immunization (dpi) with MOG35-55 peptide, lymph nodes were extracted from EAE mice. The lymphocytes were pre-treated with a range of BCP concentrations (1–100 μM) during 1 h, followed by immune stimulation with MOG35-55 (10 mg/mL) for 48 h. This BCP in vitro treatment significantly upregulated the level of IL-10 and reduced IFN-γ production after administration of MOG35-55 peptide (Figure 1). However, BCP failed to alter IL-4 levels in the lymphocytes culture after MOG35-55 peptide stimulation (Figure 1). Afterward, we evaluated whether a CB2 selective antagonist would reverse the anti-inflammatory effect shown by BCP. To conduct this experiment, lymphocytes were treated with AM630, a selective CB2 antagonist (50 μM) administered 30 min before BCP treatment, and another group of lymphocytes were treated with the selective CB2 agonist JWH-015 (50 μM) 1 h before MOG35-55. Interestingly, JWH-015 inhibited IFN-γ levels similarly to BCP treatment; however, AM630 blocked the immunomodulatory effects of BCP in the lymphocytes culture (Figure 1).

The selective agonism of CB2 receptors expressed by immune cells modulates cell migration, and decreases cytokine production [47] and antigen presentation [48]. The rank order of CB2 mRNA expression in immune cells is as follows: lymphocyte B cells > natural killer (NK) cells > monocytes > polymorphonuclear neutrophil cells > CD8+ T cells > CD4+ T cells [49]. Moreover, even though the expression of CB2 mRNA is the least significant in T lymphocytes in comparison to other major leukocyte cells, these receptors are intrinsically connected to T cell migration. In fact, the activation of CB2 receptors by multiple ligands has been shown to inhibit lymphocyte T migration responding to C-X-C motif chemokine 12 (CXCL12), a vastly characterized and effective chemokine, fundamental for T cell recruitment [50]. Taken together, these data indicate that BCP may have suppressed the encephalitogenic effects of T cells by inhibiting their recall responses (Th1 vs. Treg) and, consequently, production of pro- and anti-inflammatory cytokines, respectively. Previous data showed that BCP binds selectively to the CB2 receptor as a full agonist [12,13,14]. Additionally, CB2 receptor activation has been shown to prevent cisplatin-induced nephrotoxicity in experimental colitis, through inhibition of pro-inflammatory chemokines and infiltration of macrophage and neutrophil [31,46].

### 2.2. BCP Attenuates Disease Progression in Chronic EAE Mice Model

**Figure 2:**
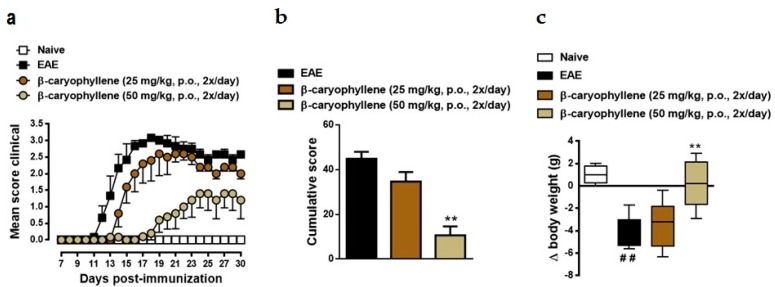
BCP treatment diminishes EAE severity and ameliorates weight loss after immunization. Daily clinical score (**a**), cumulative score (**b**), and animal body weight (**c**) were analyzed in the naive group, EAE group, and in animals under prophylactic treatment with (−)-β-caryophyllene (25 or 50 mg/kg, oral route—p.o.—daily, twice/day) during the entire experiment (from day 0 to 30 post-immunization). Data points are presented as the mean ± SEM (*n* = 6–8 animals per group and are representative of two independent experiments). ^##^ *p* < 0.001 vs. naïve group, and ** *p* < 0.001 vs. EAE-group (one-way ANOVA followed by Bonferroni’s post hoc test).

In order to assess the therapeutic potential of BCP, EAE was induced in C57BL/6 mice with myelin MOG35-55 peptide. The onset of EAE clinical symptoms was on day 11 and reached a maximum mean clinical score of 3.5 on day 19 after immunization (Figure 2). Moreover, weight loss progressed alongside EAE severity, related to both level of motor impairment and reduced food intake, as well as peak of pro-inflammatory cytokines production on the acute phase of disease. Herein, EAE mice reached their lowest weight at the peak of the disease, in comparison to the control group. These same symptoms were not observed in the naïve group (Figure 2). To evaluate the prophylactic effect of BCP treatment in the same experimental model, since day 0 of immunization, mice were treated orally with 25 and 50 mg/kg BCP (twice/day). In comparison to the untreated EAE group, BCP treatment with 25 mg/kg presented promising results, particularly at the chronic stage of disease; additionally, 50 mg/kg BCP significantly prevented motor paralysis and weight loss induced by EAE immunization (Figure 2).

**Figure 3:**
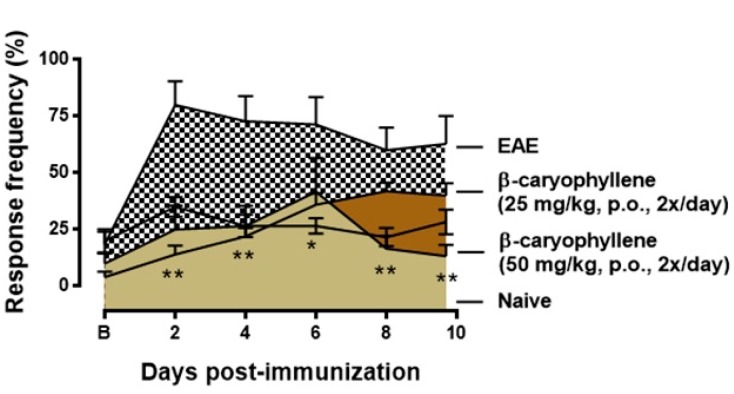
BCP treatment inhibits mechanical hiperalgesia during pre-motor phase of EAE. Treatment with (−)-β-caryophyllene (25 or 50 mg/kg, p.o., intermediate lines) or vehicle (0.9% NaCl solution—top line) was administered twice a day and throughout the test period. Naïve group—bottom line. Mechanical hiperalgesia was assessed by reduction in paw withdrawal threshold, to von Frey filament stimulation (0.4 g) at the plantar surface of the right hind paw. Data are shown as means of withdraw percentage ± SEM (*n* = 6–8 animals/group). * *p* < 0.05 and ** *p* < 0.001 vs. naïve group (one-way ANOVA followed by Newman-Keuls post hoc test).

It has been estimated that two-thirds of MS patients suffer from pain during the course of the disease at some point, whereas 40% of the patients are never pain free [51]. MS is the source of many pain syndromes; some prevail for a brief time while others are long lasting. Pain syndromes associated with MS include powerful cramps and spasms, stiffened joints, pressure pain, facial (trigeminal) pain, and other painful sensations, such as burning, itching, and shooting pain [51]. Thus, in the next set of experiments, we determined if BCP treatment could inhibit the mechanical hyperalgesia induced by EAE. In response to stimulation with von Frey filaments on the right hind paw, untreated EAE mice showed long lasting and increased frequency response (Figure 3). Treatment with BCP (50 mg/kg, p.o., twice/day) notably attenuated mechanical hyperalgesia induced by EAE immunization, especially during pre-motor phase of the disease (Figure 3). Interestingly, our data corroborates and expands previous data found in literature, which established that BCP reduced inflammatory (late phase) pain responses though the CB2 receptor in the formalin test, although it did not influence acute (early phase) responses. Furthermore, the same authors suggest that administration of BCP via oral route extenuated mechanical allodynia and thermal hyperalgesia during a neuropathic pain model, as well as reduced spinal neuroinflammation and did not induce signs of tolerance after prolonged treatment [29]. Thus, our results suggest that BCP treatment was able to extinguish clinical EAE signs, including motor impairment and symptoms induced by immunization.

These observations are corroborated by a recent report that determined that BCP showed protective effect on brain damage and chemical induced seizure through inhibition of pro-inflammatory cytokines productions, such as TNF-α and IL-1β, as well as restored the activity of catalase (CAT), superoxide dismutase (SOD), and glutathione peroxidase (GPx) [52]. Likewise, Cheng and colleagues demonstrated that BCP, given orally, activated the CB2 receptor and the peroxisome proliferator-activated receptor-γ (PPARγ) pathway, preventing the Alzheimer-like phenotype in APP/PS1 mice. Furthermore, this cognitive effect was positively related with decreased β-amyloid load in the cerebral cortex and hippocampus. In addition, BCP treatment inhibited the levels of COX-2 protein as well as glial activation, and the mRNA levels of the pro-inflammatory cytokines in the cerebral cortex [27]. Hence, to establish the status of these cells and inflammatory mediators in BCP-treated EAE mice, we evaluated glial activation, oxidative damage, and demyelination in brain tissues from both untreated and BCP-treated EAE mice.

### 2.3. BCP Inhibits Glial Activation, Oxidative Damage, and Demyelination during EAE Development

**Figure 4:**
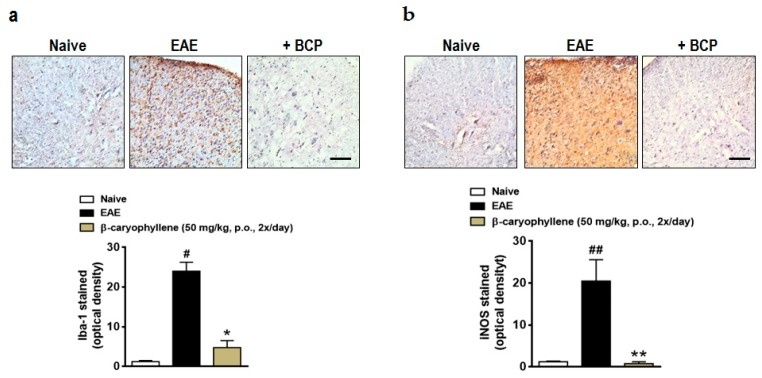
BCP treatment prevents microglial activation and oxidative damage in the CNS after EAE induction. C57BL/J6 mice were immunized with MOG35-55 and administered BCP (50 mg/kg) daily p.o. throughout the whole experiment (from day 0 to 30 post-immunization). (**a**) Glial activation marker (Iba-1) and (**b**) oxidative damage (iNOS) were measured in the lumbar spinal cord. Scale bar corresponds to 50 μm and applies throughout. Data are presented as mean ± SEM (*n* = 5 animals per group and are representative of two independent experiments). ^#^ *p* < 0.05 and ^##^ *p* < 0.001 vs. naïve group; * *p* < 0.05 and ** *p* < 0.001 vs. vehicle-treated EAE mice (one-way ANOVA followed by Newman–Keuls post hoc test).

**Figure 5:**
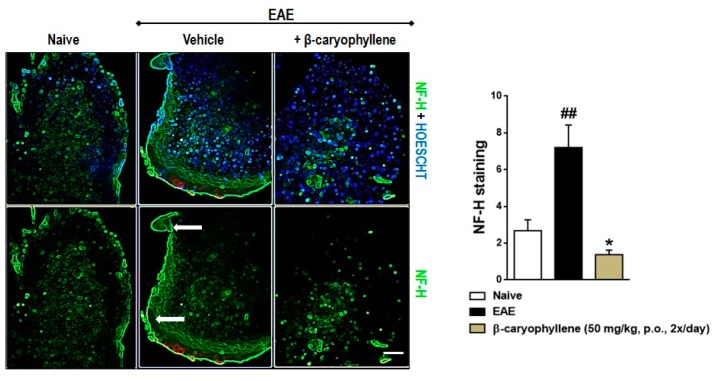
BCP treatment inhibits demyelination in the CNS during EAE pathology. C57BL/J6 mice were subjected to immunization with MOG35-55 and given BCP (50 mg/kg) daily p.o. throughout the whole experiment (from day 0 to 30 post-immunization). Neurofilament-H (green color) was measured in the lumbar spinal cord. Hoechst 33342 dye was used for nuclei stain—blue color. Scale bar corresponds to 50 μm and applies throughout. Arrow indicates demyelination area—white matter region. Data are presented as the mean ± SEM (*n* = 5 animals per group). ^##^ *p* < 0.001 vs. naïve group and * *p* < 0.05 vs. vehicle-treated EAE mice (one-way ANOVA followed by Newman–Keuls post hoc test).

**Figure 6:**
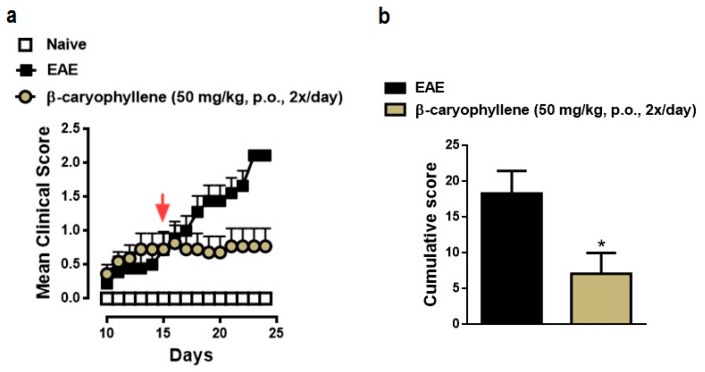
BCP therapeutic treatment prevents the clinical course of EAE. Daily clinical score (**a**) and cumulative score (**b**) of the vehicle- and BCP-treated EAE mice. C57BL/J6 mice were immunized with MOG35-55 and administered BCP (25 or 50 mg/kg) daily p.o. from day 0 until the end of the experiment (25 days post-immunization). Arrow indicates when treatment began. Data points are presented as the mean ± SEM (*n* = 6–8 animals per group and are representative of two independent experiments). * *p* < 0.05 vs. vehicle-treated EAE mice (one-way ANOVA followed by Bonferroni’s post hoc test).

Therefore, in order to further assay the mechanisms of action of BCP, in a new set of experiments, the expression of ionized calcium binding adaptor molecule 1 (Iba-1), inducible nitric oxide synthase (iNOS), and neurofilament-H was measured in the lumbar spinal cord of EAE-untreated and treated mice. There was a significant increase in the expression of Iba-1 and iNOS in the lumbar spinal cord of the EAE-control group (Figure 4). Moreover, the expression of neurofilament-H, a demyelination marker, was increasingly expressed by EAE immunization in the control group (Figure 5). Relevantly, BCP (50 mg/kg, p.o.) treatment downregulated the microglial activation, oxidative damage (Figure 5), and, consequently, demyelination (Figure 6). This finding validates and expands previous data demonstrating that through activation of BV2 microglia following hypoxic exposure, BCP inhibited cytotoxicity by decreasing the release of pro-inflammatory cytokines, including TNF-α, IL-6, and IL-1β. Additionally, the same authors demonstrated that BCP downregulated the activation of nuclear factor κB (NF-κB), and CB2R small RNA interference completely abolished these actions [28]. Altogether, our research indicates that BCP selectively increases the infiltration/differentiation of Treg and inhibits Th1 myelin-specific cells in the CNS through activation of the CB2 cannabinoid receptor. These results are in agreement with Bento and colleagues, who demonstrated that AM630, a CB2 antagonist, reverted the inhibitory effect of BCP on pro-inflammatory chemokine CINC-1/CXCL1 levels and mRNA expression of TNF-α in the IEC-6 cell line. In addition, the same authors showed that the therapeutic effects of BCP on experimental colitis were reversed by concomitant administration of CB2 and PPAR-γ antagonists [46]. Thus, further investigation is required to verify whether BCP modulates the course of EAE through direct interaction with CB2 receptor or endogenous CB pathways, such as activation of peroxisome proliferator-activated receptor-γ (PPARγ).

### 2.4. BCP Therapeutic Treatment Inhibits Progression the Clinical Signs of EAE

The observed anti-inflammatory action of BCP on the induction phase of EAE led us to further examine if commencement of therapy after onset of disease, during chronic phase, could also exert therapeutic response. In order to explore this hypothesis, BCP was given to ongoing active EAE in C57BL/6 mice, starting after mice had already presented visible clinical symptoms (score of 1.5 ± 0.5) of disease (on day 15). BCP at 50 mg/kg administered twice daily notably blocked the clinical severity of the disease, indicating maximal inhibition of 62% based on the cumulative score (Figure 6).

### 2.5. BCP Prophylactic Treatment Downregulated CD4+ and CD8+ T Lymphocytes

**Figure 7:**
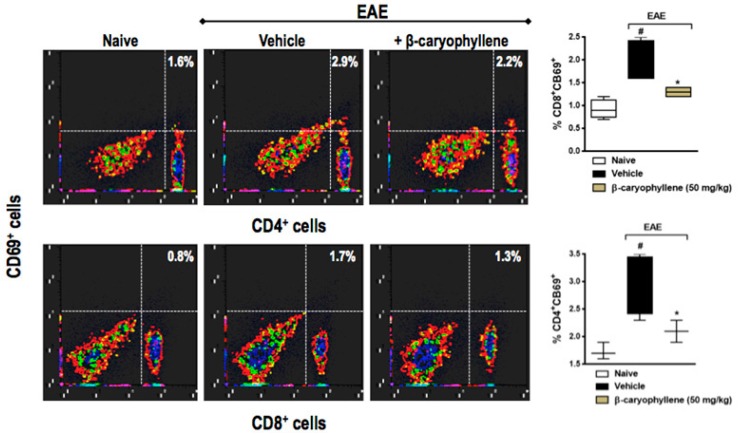
Preventive treatment with β-caryophyllene reduced the population of CD8+ (top panel) and CD4+ (bottom panel) T lymphocytes in inguinal lymph nodes during EAE pathology. C57BL/J6 mice were immunized with MOG35-55 and given BCP (50 mg/kg) daily p.o. from day 0 until the end of the experiment (30 days post-immunization). Cells were analyzed by flow cytometry—1 × 10^6^ cells. Data are presented as the mean ± SEM (*n* = 6–8 animals per group). ^#^ *p* < 0.05 vs. naive group, and * *p* < 0.05 vs. vehicle-treated EAE mice (one-way ANOVA followed by Bonferroni’s post hoc test).

**Figure 8:**
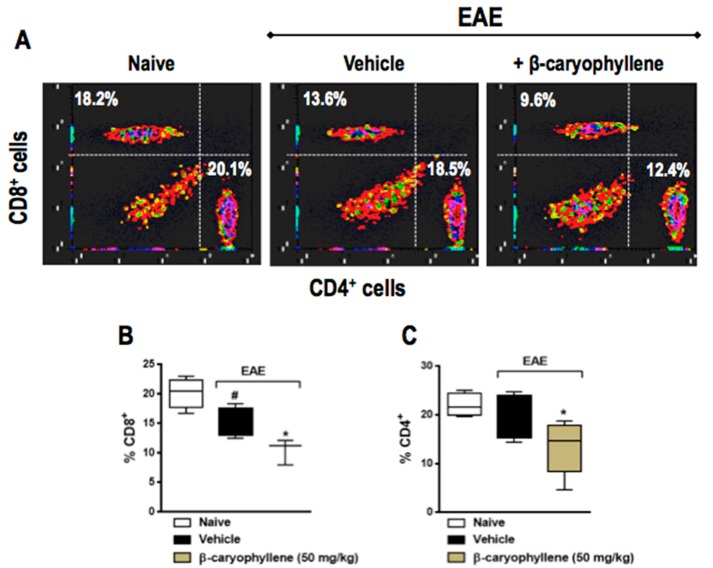
Preventive treatment with β-caryophyllene inhibited CD8+CD69+ (**A**,**B**) and CD4+CD69+ (**A**,**C**) T lymphocytes activation in inguinal lymph nodes during EAE pathology. C57BL/J6 mice were immunized with MOG35-55 and given BCP (50 mg/kg) daily p.o. from day 0 until the end of the experiment (30 days post-immunization). Cells were analyzed by flow cytometry—1 × 10^6^ cells. Data are presented as the mean ± SEM (*n* = 6–8 animals per group). ^#^ *p* < 0.05 vs. naive group, and * *p* < 0.05 vs. vehicle-treated EAE mice (one-way ANOVA followed by Bonferroni’s post hoc test).

Since CD4+ Th-induced inflammation is thought to be responsible for the neurodegenerative component of chronic EAE, we asked whether BCP would interfere with CD4+ and CD8+ T cells. The percentage of CD4+ and CD8+ T cells was significantly lower in EAE, moreover, both CD4+ and CD8+ T cells were significantly decreased in BCP prophylactically treated animals (Figure 7). In order to evaluate activation of T lymphocytes, the co-expression of CD69 cellular marker was measured through the double staining of CD4+CD69+ and CD8+CD69+. In fact, the population of CD4+CD69+ and CD8+CD69+ T lymphocytes were remarkably increased in EAE-untreated animals when assessed 25dpi, when compared with control naive mice. Furthermore, BCP treatment (50 mg/kg, p.o.) inhibited both CD4+ and CD8+ T cells, as well as their activation in the peripheral lymphoid tissue during EAE development (Figure 8).

## 3. Materials and Methods

### 3.1. Chemicals and Reagents

BCP (catalogue Nr. 22075), bovine serum albumin (BSA), deoxyribonuclease I (DNase), H2O2, Incomplete Freund’s adjuvant oil, Na-ethylenediamine tetraacetic acid (EDTA), penicillin, *Pertussis* toxin, streptomycin, and trypsin were obtained from Sigma Chemical Co. (St. Louis, MO, USA). The RPMI1640 and fetal bovine serum were purchased from GIBCO (Carlsbad, CA, USA). *Mycobacterium tuberculosis* extract H37 Ra was purchased from Difco Laboratories (Detroit, MI, USA). Anti-mouse IL-10, IL-4, and IFN-γ ELISA MAX Deluxe Sets were obtained from BioLegend (San Diego, CA, USA). MOG35-55 peptide (MEVGWYRSPFSRVVHLYRNGK), myelin oligodendrocytes glycoprotein, was obtained from EZ Biolab, Carmel, IN 46032, USA. AM630 and JWH-015 were obtained from Tocris Bioscience (Ellisville, MO, USA). Anti-CD4 and anti-CD8 obtained from BD Pharmingen (San Jose, CA, USA). Primary and secondary antibodies were purchased from Santa Cruz Biotechnology^®^ (Santa Cruz, CA, USA).

### 3.2. Animals

C57BL/6 female mice (6 to 12 weeks old) from Laboratório de Autoimunidade e Imunofarmacologia (LAIF), Universidade Federal de Santa Catarina, were arranged in groups of four to six animals per cage, at 22 ± 1 °C, under a 12-h light/dark cycle (lights on at 7:00 a.m.) with food and water provided ad libitum. Every procedure hereby presented was followed according to international ethic guidelines of “Principles of laboratory animal care” (National Institutes of Health, NIH publication no. 85-23). This experiment was approved by Animal Ethics Committee of the Universidade Federal de Santa Catarina (CEUA-UFSC) under the protocol number PP00956—date 8 December 2014.

### 3.3. Active EAE Induction in C57BL/6 Mice

Chronic progressive disease was induced in mice with MOG35-55 peptide. In sum, mice were injected in the flank region with 200 μL of an inoculum containing 200 μg of MOG35-55 peptide emulsified in incomplete Freund’s adjuvant (IFA) containing 0.5 mg *Mycobacterium tuberculosis* H37Ra. MOG-sensitized mice were injected intraperitoneally (i.p.) with 300 ng of *Pertussis* toxin on the day of the immunization and after 2 days.

### 3.4. Clinical Evaluation

Mice were submitted to daily clinical evaluation in a double-blind manner in order to assess signs of EAE for up to 25–30 days after immunization. They were examined, weighed, and scored on a scale of 0–5 according to the clinical severity of their neurological symptoms as follows: score 0, no abnormality; score 0.5, partial loss/reduced tail tone, assessed by inability to curl the distal end of the tail; score 1, tail atony; score 1.5, slightly/moderately clumsy gait, impaired righting ability, or combination; score 2, hind limb weakness; score 2.5, partial hind limb paralysis; score 3, complete hind limb paralysis; score 3.5, complete hind limb paralysis and fore limb weakness; score 4, tetraplegia; and score 5, moribund or death.

### 3.5. Treatment Procedure

Mice were treated daily with 25 or 50 mg/kg BCP (5% Tween 80 in saline 0.9% NaCl solution) by oral route—gavage (p.o.)—twice a day, after immunization, prophylactically (from day 0, before the onset of clinical symptoms) or therapeutically (from day 15, after the onset of the symptoms) until end of the experiment. Treatments with cannabinoid 2 receptor (CB2) agonist, JWH-015 (50 μM), was used during ex vivo assay. In vitro, BCP treatments (1–100 μM) were administered, in the absence and presence of the selective CB2 antagonist, AM630 (50 μM), administered 30 min before BCP treatment. The dose of each drug was chosen based on preliminary studies [46,53,54,55].

### 3.6. Isolation of Lymphocytes

The 10-week-old EAE C57BL/6 female mice were euthanized and inguinal lymph nodes were removed. The lymph nodes were lysed to prepare as single-cell suspensions. The cells were washed twice and resuspended in RPMI 1640 with the concentration adjusted to 1 × 10^6^ cells/mL total lymphocytes. The lymphocytes were pretreated with a range of concentrations of BCP (1–100 μM) for 1 h followed by stimulation with MOG35-55 (10 μg/mL) for 48 h. The production of immune mediators was determined by ELISA.

### 3.7. Flow Cytometry Assay

Herein, we performed CD4+ and CD8+ T cells quantification by flow cytometry. Inguinal lymph nodes (LN) obtained from each animal group were macerated in RPMI 1640 medium and filtered through a 220 μm filter. The resulting suspension was centrifuged at 1500× *g* for 7 min, then supernatant was discarded, and the cell pellet was resuspended in RPMI 1640 medium supplemented with 10% fetal bovine serum, 20 mM HEPES, 3 × 10^−5^ M 2-mercaptoethanol, 100 U/mL penicillin, and 100 mg/mL streptomycin. The cells were incubated with the following antibodies for 20 min at 4 °C: anti-CD4-PerCP, anti-CD8a-APC, and anti-CD69-PE. The data were collected with FACS Canto II (BD Biosciences, San Jose, CA, USA) and analyzed by means of FlowJo (version 7.5, FlowJo, LLC, Ashland, OR, USA) software.

### 3.8. Histology

To assess the microglial activation, oxidative response and demyelination in the CNS, treated and untreated groups of C57BL/6 mice were euthanized at the chronic stage of EAE disease (day 30) by CO_2_ asphyxiation. Their spinal cords of the lumbar region were removed and fixed in 10% buffered formalin. After paraffin embedding, transverse sections of spinal cord of 12 μm-thick (nine sections per animal) were stained with Iba-1, iNOS, and NF-H, respectively. The spinal cord sections from five representative mice (BCP treated and untreated) were processed and stained with Hoechst 33342 dye (for nuclei stain). A Q imaging digital camera connected to an Olympus Bx4 microscope was used. The immunostaining was assessed at four levels of the lumbar spinal cord. Specifically, four alternate 5-μm sections of the lumbar spinal cord with an individual distance of 150 μm were obtained between L4 and L6. A threshold optical density that best discriminated staining from the background was obtained using the NIH ImageJ 1.36b imaging software (NIH, Bethesda, MD, USA). We captured four images of each spinal cord subregion (dorsal, ventral, lateral, central) per section (eight images per section and 32 images per mouse, *n* = 5 animals/group). Then, the total pixel intensity was determined and the data were expressed as optical density (O.D.).

### 3.9. Mechanical Hyperalgesia

Mice were assessed for mechanical withdrawal thresholds in an individual Plexiglas housing (9 cm × 7 cm × 11 cm) with wire mesh floor, and given time to adjust to the ambiance while exploring and grooming until settling down. A von Frey filament—0.4 g (Stoeling Company, Wood Dale, IL, USA) was handled in an ascending movement in order to pressure the plantar surface of the hind-paw. The bending force of the von Frey filament triggering the withdrawal of the hind-paw was recorded. Hind-paw withdrawal was considered as positive response. Each hind-paw was measured three times and the average values from the three measurements were recorded as the mechanical paw withdrawal threshold. Animals were evaluated until day 10 post-immunization, before onset of motor dysfunctions.

### 3.10. Statistical Analysis

Statistical analysis was performed with the GraphPad Prism 6 software (San Diego, CA, USA) by analysis of variance (ANOVA). Post hoc analyses were executed by the Bonferroni’s multiple comparison test or Newman–Keuls test. Results are expressed as mean ± standard error of the mean (SEM); *p* < 0.05 and *p* < 0.001 were considered statistically significant.

## 4. Conclusions

Altogether, a dietary non-psychoactive CB2 receptor ligand sesquiterpene BCP preventively or therapeutically blocked the development and progression of clinical and neurological signs of EAE, which was correlated with the hindrance of immune cell activation, neuroinflammation, and demyelinating processes in the CNS. Moreover, our findings also demonstrate that the effect of BCP on EAE most likely occurs due to the activation of the CB2 receptor, resulting in a corresponding downregulation of the migration of encephalitogenic T cells into the CNS, which in turn may increase Treg vs. inhibit Th1 polarization and expression. Furthermore, our data establishes that BCP constitutes an alluring therapeutic molecule towards the treatment of MS, along with other autoimmune diseases.
